# Severe Fever with Thrombocytopenia Syndrome in Japan and Public Health Communication

**DOI:** 10.3201/eid2103.140831

**Published:** 2015-03

**Authors:** Tomoya Saito, Kazuko Fukushima, Kazunori Umeki, Kensuke Nakajima

**Affiliations:** Health Service Bureau, Ministry of Health, Labour and Welfare, Tokyo, Japan (T. Saito, K. Fukushima, K. Umeki, K. Nakajima);; National Institute of Public Health, Saitama, Japan (T. Saito)

**Keywords:** Phlebovirus, public health, communicable diseases, viruses, Japan, communication

## Abstract

A fatal case of severe fever with thrombocytopenia syndrome was reported in Japan in 2013. The ensuing process of public communication offers lessons on how to balance public health needs with patient privacy and highlights the importance of multilateral collaborations between scientific and political communities.

Severe fever with thrombocytopenia syndrome (SFTS), caused by SFTS virus (SFTSV), was initially identified in China in 2011 (*1*). SFTS manifests with fever, vomiting, and diarrhea accompanied by clinical signs including low platelet and leukocyte counts; the illness can be fatal. The SFTSV vectors, *Haemaphysalis longicornis* and *Rhipicephalus microplus* ticks, inhabit Japan, but the virus had not been detected in ticks in this country, nor had there been a case report of SFTS from Japan. Thus, the public health risk from SFTS was not recognized until a fatal case of SFTS was confirmed in Japan in early 2013 (*2*,*3*). 

## The Case

At the end of December 2012, a virologist successfully isolated an unknown virus from a clinical sample from a person in Japan who had died of unknown causes. Whole-genome sequencing showed that the isolate’s gene sequences were highly similar to those of SFTSV (*4*). The SFTS diagnosis was confirmed by the Japan National Institute of Infectious Diseases (NIID) on January 29, 2013. 

On January 30, a rapid communication on the website of the Infectious Agents Surveillance Report (IASR) described this case as the first case of SFTS in Japan (*2*). In addition, health officials from Yamaguchi Prefecture, where the patient resided, and from the Ministry of Health, Labour and Welfare (MHLW) announced the identification at a press conference. On the same day, the MHLW issued a notice requesting that medical doctors voluntarily report suspected cases that fulfilled the interim SFTS case definition (Table). From that date through March 31, a total of 23 suspected cases were reported, blood samples were submitted, and a retrospective study was conducted (*5*). NIID testing confirmed 11 SFTS cases (median patient age 71 years, range 50–84 years), including a case from 2005. Officials from prefectures in which cases were confirmed made public announcements for most of these cases, and the MHLW issued a press release to convey the information nationwide. Clinical details of retrospective cases were reported on the IASR website (*6**,**7*).

**Table:** Interim case definition for retrospective or prospective voluntary case reports of suspected severe fever with thrombocytopenia syndrome, Japan*

Criteria
Fever >38°C
Gastrointestinal tract symptoms (e.g., nausea, vomiting, abdominal pain, diarrhea, melena)
Thrombocytopenia, <100 × 10^9^ platelets/L
Leucopenia, <4 × 10^9^ leukocytes/L
Elevated levels of aspartate aminotransferase, alanine aminotransferase, and lactate dehydrogenase
Absence of other causes
Death or admission to an intensive care unit because of symptoms

*All criteria required for case confirmation.

Effective March 4, 2013, the MHLW revised the Order for Enforcement of the Infectious Disease Control Act to include SFTS as a class IV infectious disease. These diseases are notifiable, but mandatory hospitalization or restrictions of a patient’s activities are not warranted. After that date, the MHLW began to report only the case numbers in each prefecture in the Infectious Disease Weekly Report. New infections were reported starting in April 2013. Descriptions of clinical manifestations for new case-patients were also shared promptly on the IASR website (*8**–**10*).

NIID developed a reverse transcription PCR to detect the Japanese strain of SFTSV, and PCR primers and reagents were distributed from NIID to 79 local public health laboratories by the end of March 2013 to establish countrywide diagnostic capacity. Laboratory results were initially confirmed by NIID, acting in a reference capacity for local laboratories (*11*). 

To investigate the epidemiology, pathology, life cycle, countermeasures, and geographic distribution of SFTSV in Japan, the government established a 3-year research project in May 2013. Japan has 47 indigenous tick species, but the specific vectors of SFTSV and their habitats, proportion of virus carriers, and interactions with wild animals are unknown. Systems were developed to detect the SFTSV gene in ticks and SFTS antibodies in animal blood samples (*8*). Results of an interim investigation of ticks and of blood samples from hunting dogs, wild deer, and wild boar suggest that, despite the limited reports of human cases from the western part of Japan, the geographic distribution of virus is more extensive than previously understood (*12**,**13*). *H. longicornis* and *Amblyomma testudinarium* ticks were identified as SFTSV vectors in Japan; however, other species may also be carriers of the virus (*13*).

The initial case of SFTS in Japan attracted substantial public attention, creating the challenge of balancing public health needs with the protection of patient privacy. One of the basic philosophies of the Infectious Disease Control Act is the proactive disclosure of information on the situation, trends, cause, and disclosure of information necessary for prevention and treatment of infectious diseases. At the same time, the Minister of Health, Labour and Welfare and the governors of local governments must remain mindful of the protection of personal information when disclosing information about cases (Paragraph 2, Article 16, Infectious Disease Control Act). The amount of personal identifying information to disclose must be evaluated on a case-by-case basis and must be consistent with the philosophy of the Act. Some public health plans, such as the Smallpox Preparedness Guideline (*14*), include a press release template, which includes disclosure of sex, age, and municipal area of residence. However, the risk communication guidelines in the Guidelines for Pandemic and New Infectious Diseases do not specify the items of patient information to be disclosed, although, in principle, the area of patient’s residence at a municipal level should be disclosed to inform the public of the area(s) in which human-to-human spread is a risk (*15*).

Disclosure of personal information for the initial SFTS case-patient was limited to sex, adult status, and prefecture of residence; this information was limited to respect the wishes of the bereaved family. Age, municipal area of residence, and date of death were not disclosed because of the risk that these variables would result in patient identification. However, the amount of information disclosed about the initial case-patient was criticized at the House of Representatives Budget Committee meeting on April 2, 2013. A House of Representatives member claimed that more detailed information about the patient’s area of residence was needed so that the geographic area of risk could be identified and residents could be alerted. The Minister responded by saying that the risk for SFTSV infection was not limited to the area surrounding the patient’s residence and that tick bite precautions should be practiced throughout the country.

The case information disclosure policy was tempered as public attention waned; after the first 5 reported cases, the patient’s age category was disclosed. Overall, the proportion of publicized SFTSV case reports, including the area of residence of patients at a municipal level, increased from 4/12 (30%) for cases occurring before 2012 to 24/40 (60%) for cases occurring during 2013.

## Conclusions

A total of 40 SFTS cases were reported in Japan during 2013, which suggests that underdiagnosis might have occurred before the index case was identified. The success of the initial diagnosis of SFTS in the country was the result of a persistent investigation of a death by unknown causes conducted by hospital clinicians who were not aware that the patient had a history of a tick bite. However, the clinicians consulted an animal viral disease expert for assistance in isolating the causative agent because the clinical picture was a virus-associated hemophagocytic syndrome (Dr. Takahashi and Dr. Ishido, pers. comm.). 

Our report shows that multilateral collaborations among investigators, including medical infectious disease specialists, epidemiologists, pathologists, virologists, entomologists, and veterinarians, were required for a timely response to this emerging vector-borne disease. Cooperation from professional organizations such as Dainihon Ryoyukai, a hunters’ organization, was crucial for obtaining blood samples from wild animals. Maximizing the use of local government resources is essential for a prompt national investigation (e.g., tick collection and blood sample collection from wild animals). Further collaborative investigation is expected to lead to a better understanding of SFTS and SFTSV in Japan.
